# Trans-spliced Cas9 allows cleavage of HBB and CCR5 genes in human cells using compact expression cassettes

**DOI:** 10.1038/srep10777

**Published:** 2015-07-01

**Authors:** Eli J. Fine, Caleb M. Appleton, Douglas E. White, Matthew T. Brown, Harshavardhan Deshmukh, Melissa L. Kemp, Gang Bao

**Affiliations:** 1 Department of Biomedical Engineering, Georgia Institute of Technology and Emory University, Atlanta, GA, 30332, USA

## Abstract

CRISPR/Cas9 systems have been used in a wide variety of biological studies; however, the large size of CRISPR/Cas9 presents challenges in packaging it within adeno-associated viruses (AAVs) for clinical applications. We identified a two-cassette system expressing pieces of the *S. pyogenes* Cas9 (SpCas9) protein which splice together *in cellula* to form a functional protein capable of site-specific DNA cleavage. With specific CRISPR guide strands, we demonstrated the efficacy of this system in cleaving the *HBB* and *CCR5* genes in human HEK-293T cells as a single Cas9 and as a pair of Cas9 nickases. The trans-spliced SpCas9 (tsSpCas9) displayed ~35% of the nuclease activity compared with the wild-type SpCas9 (wtSpCas9) at standard transfection doses, but had substantially decreased activity at lower dosing levels. The greatly reduced open reading frame length of the tsSpCas9 relative to wtSpCas9 potentially allows for more complex and longer genetic elements to be packaged into an AAV vector including tissue-specific promoters, multiplexed guide RNA expression, and effector domain fusions to SpCas9. For unknown reasons, the tsSpCas9 system did not work in all cell types tested. The use of protein trans-splicing may help facilitate exciting new avenues of research and therapeutic applications through AAV-based delivery of CRISPR/Cas9 systems.

Although systems using clustered regularly interspaced palindromic repeats (CRISPR) combined with the Cas9 nuclease have been used in many model organisms and cell lines to either modify a targeted sequence of DNA^1^,^2^,^3^,^4^, regulate gene expression^5^,^6^, or modify epigenetic markers^7^, several challenges remain in translating this promising technology into human therapeutics. A major advantage of CRISPR/Cas9 systems is their simplicity; by redesigning a short piece of RNA, known as a single guide RNA (sgRNA), the Cas9 protein can be guided to nearly any region of interest in the genome. However, in advancing CRISPR/Cas9-based therapeutics, the large size of Cas9 remains a challenge for viral-based *in vivo* delivery^8^. While delivery of a human codon-optimized form of the *S. pyogenes* Cas9 (SpCas9) via lentiviruses has been achieved^9^, a more relevant therapeutic platform is adeno-associated viruses (AAVs) which would allow for *in vivo* delivery in humans with reduced possibility of triggering immune responses^10^. However, AAVs have much stricter packaging limits than lentiviruses and cannot function efficiently if the size of the packaged DNA is >4.8 kb^11^. This is problematic for packaging SpCas9 since expression of SpCas9 with standard sized promoter and polyadenylation elements would require >5.3 kb of DNA (Fig. 1a). The recently solved SpCas9 crystal structure revealed that both the amino and carboxyl termini of SpCas9 contain critical domains^12^, and attempts to truncate the ends of SpCas9 abolished all functional activity^13^. Although the structure of SpCas9 showed that the REC2 domain is not critical to SpCas9 function and could be deleted at a cost of a 50% reduction in DNA cleavage activity^12^, the deletion of REC2 does not shorten SpCas9 appreciably (Fig. 1b). Recent work with minimal promoter and polyadenylation sequences has produced functional SpCas9 constructs that can be effectively packaged in AAVs^14^,^15^, but such constraints on promoter selection will limit their application. Furthermore, as noted by Mali *et al*.^8^, the potential of Cas9 lies not only in its ability as a nuclease, but also in its ability to be catalytically inactivated (known as “dead” or dCas9) and fused with other functional domains at either the N-terminus or C-terminus in order to regulate gene expression, epigenetic modifications, or more precise DNA cleavage^3^,^4^,^5^,^6^,^13^. However, fusing effector domains to Cas9 further increases the size of the protein thereby further increasing the degree of difficulty of packaging it in an AAV vector.

An approach that had some success in delivering large proteins via AAVs is intein-mediated protein *trans*-splicing^16^. An intein is a protein sequence analogous to an mRNA intron; after the protein is expressed, the intein sequence self-catalytically splices itself out, joining the flanking “extein” (analogous to mRNA exons) sequences together with a peptide bond. Certain inteins are able to be split into two fragments which can be expressed separately (in *trans*), and subsequently associate with each other and still splice the two flanking extein sequences together. We hypothesized that SpCas9 might be able to be split and re-joined using this process (Fig. 1d). After testing different inteins and split points within SpCas9, we identified a system using the gyrase A (GyrA) intein from *Mycobacterium xenopi*^17^ which robustly recapitulates the nuclease activity of the wild-type CRISPR/Cas9 system^2^. Our *trans*-splicing system consists of two cassettes (created by modifying the pX330 plasmid^2^), each less than 4 kb, which express two single guide RNAs (sgRNAs) and the two halves of SpCas9 fused to the split portions of the Mxe GyrA intein (Fig. 1c). Sin**c**e the *trans*-splicing approach dramatically reduces the length of protein coding sequence each cassette is required to contain, there is ample room remaining to add sequences in future studies that allow expression of additional sgRNAs (which have been shown to promote highly synergistic effects^5^,^6^) and fusion of effector domains with dCas9 (Supplemental Figure S1). Effector domains that are small enough to be used in our system include FokI (nuclease)^3^,^4^, VP64 (gene upregulation)^5^,^6^, KRAB (gene repression)^13^, and SID4X (histone deacetylation)^7^, all of which have been previously fused successfully to engineered DNA binding domains.

## Results

### Developing *trans*-splicing SpCas9

In our initial experiments to design the system, several different inteins that had previously been shown to be able to be split apart and mediate protein *trans*-splicing were considered. Inteins require certain amino acids in the +1 extein position (the amino acid flanking the C-terminus of the intein) to complete the self-catalytic splicing reaction, typically with either a hydroxyl or thiol reactive group (i.e. cysteine, serine, or threonine). Additionally, the -1 extein position (the amino acid flanking the N-terminus of the intein) is also typically preserved in engineered trans-splicing experiments in order to increase the chance that splicing will be successful^18^. Therefore, we examined SpCas9 for the presence of specific amino acid di-residue motifs; “YT” for the *M. xenopi* gyrase A (Mxe GyrA) intein^17^, “GS” for the *Synechocystis* species DNA B polymerase subunit (Ssp DnaB) intein^17^, “YC” for the *Synechocystis* species DNA E polymerase subunit (Ssp DnaE) intein^19^, and “GC” for the *S. cerevisiae* Vacuolar ATPase (Sce VMA) intein^20^. SpCas9 contains no “YC” or “GC” motifs, so the Ssp DnaE and Sce VMA inteins were not considered further.

For Mxe GyrA, three “YT” motifs exist (Y72, Y656, and Y1336), but only Y656 results in an appreciable reduction in the size of the largest split piece of Cas9, so only this split point was considered further. For Ssp DnaB, four “GS” motifs exist (G408, G729, G792, and G1247). G408 and G1247 would not result in appreciable Cas9 size reduction. While both G729 and G792 would allow development of delivery cassettes less than 4.8 kb, G729 would allow for a more even split of the Cas9 protein and was chosen for investigation. Because the Ssp DnaB G729 failed in initial testing and the Mxe GyrA Y656 showed very promising initial results (Supplementary Figure S2), the Ssp DnaB G792 formation was not tested.

In an effort to determine why the GyrA trans-splicing system successfully displayed DNA cleavage activity while the DnaB trans-splicing system failed, we examined the recently published crystal structure of SpCas9^12^. Using VMD^21^, we visualized the crystal structure of SpCas9. At the split point used in the GyrA system (Y656), the split point is in a relatively unstructured region between an alpha helix and a beta sheet (Fig. 2a). Additionally, the linker region C-terminal of the split point has two glycine residues (highlighted in bold) which may further contribute to the flexibility of this region. We hypothesize that because this region of SpCas9 is relatively flexible and unstructured, it may have been more amenable to being expressed in *trans* and spliced back together. While the split point used in the DnaB intein system (G729) is also in a linker between two structured regions (Fig. 2b), this linker contains a proline residue (highlighted in bold), which may be structurally important in forming a tight bend between these two alpha helices. We hypothesize that because this region of SpCas9 may be more structurally important, it is possible that the protein may not have been able to re-fold correctly after splicing. Because several other Cas9 orthologs from different species have shown promise as genome engineering tools^22^, we examined whether there might be homologs to the Y656 split point used in the SpCas9 GyrA system. Unfortunately, BLAST^23^ alignment to the *N. meningitidis* Cas9 (NmCas9) (Fig. 2c) and *S. thermophilus* Cas9 (StCas9) (Fig. 2d) found that almost no homology to the region surrounding the Y656 SpCas9 split point, suggesting that this exact location would not be translatable to these other Cas9 proteins. However, many potential split points were found in these proteins using the amino acid motifs outlined in the approach above, suggesting that it may be possible to create *trans*-splicing systems for those Cas9 proteins as well. Further, just as it has been shown that Cas9 proteins from different species are orthogonal, many split inteins have been shown to be orthogonal as well^20^, suggesting that trans-splicing systems could be created to simultaneously deploy orthogonal split Cas9 proteins which would only splice together with the corresponding Cas9 piece via orthogonal split inteins.

### Nuclease activity of *trans*-splicing SpCas9 in HEK-293T cells

**Figure 1:**
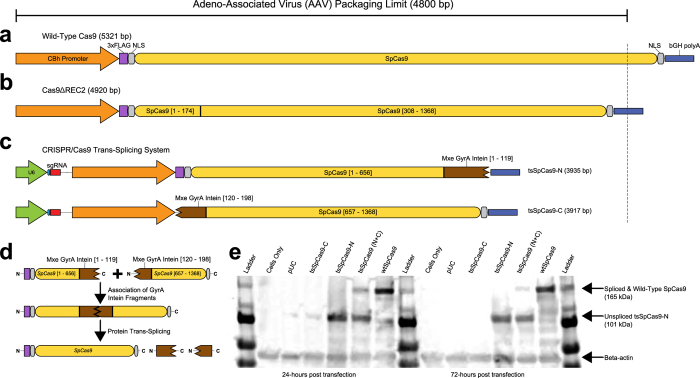
Depiction of the wild-type and trans-splicing Cas9 systems. (**a**) Size of genetic components used for expression of wild-type Cas9^2^. (**b**) Components used for a deletion mutant of Cas9^12^. (**c**) The two cassettes necessary for the trans-splicing Cas9 system. (**d**) Depiction of the proteins of the trans-splicing system forming a functional wild-type Cas9 after being expressed in cells. (**e**) Western blotting for the 3xFLAG epitope present on tsSpCas9-N and wtSpCas9 in 293T cells.

**Figure 2:**
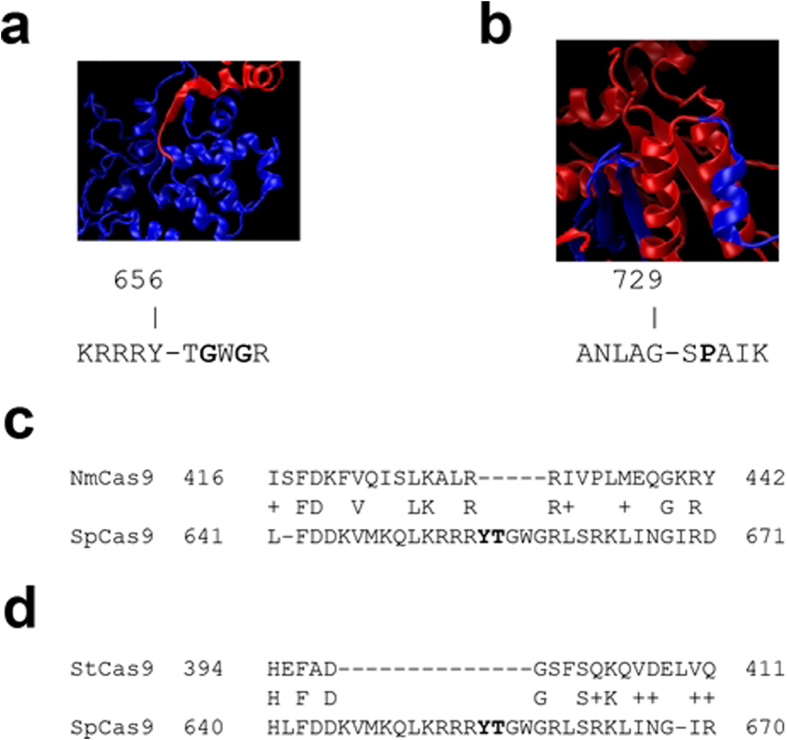
SpCas9 split points and comparison to other Cas9 orthologs. The crystal structure of SpCas9 at the split points tested for the GyrA intein (**a**) and DnaB intein (**b**) are shown with the portion of SpCas9 N-terminal to the split point shown in blue and the portion C-terminal of the split point shown in red. The amino acids flanking the split point (designated by a hyphen) are provided. BLAST alignments of the region in SpCas9 surrounding the GyrA intein split point to *N. meningitidis* Cas9 (**c**) and to *S. thermophilus* Cas9 (**d**).

**Figure 3:**
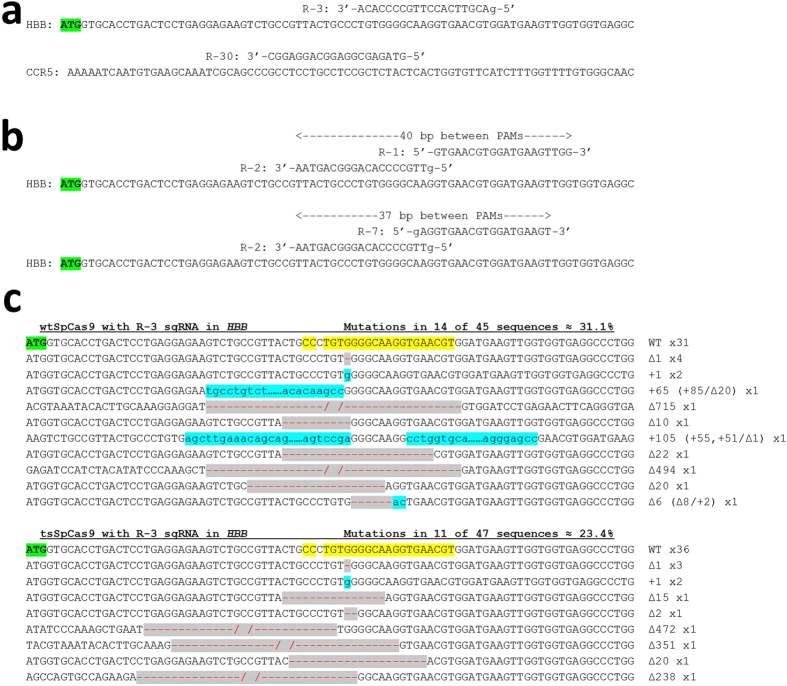
sgRNA orientations and TOPO sequencing results. (**a**) Orientation of sgRNAs targeting *HBB* and *CCR5* tested with a single Cas9 nuclease. The start codon of *HBB* is bolded and highlighted in green. Mismatched 5’ bases in sgRNAs are denoted by lowercase letters. (**b**) Orientation of sgRNAs targeting HBB tested together as a pair of Cas9 nickases. The distances between the PAMs of both sgRNAs are denoted. (**c**) Results of TOPO sequencing from transfections of the R-3 sgRNA with wtSpCas9 or tsSpCas9. The nuclease target is highlighted in yellow in the wild-type (WT) sequence. In mutated sequences, deletions are denoted by a red hyphen highlighted in grey and insertions are denoted by lowercase text highlighted in blue. The size of the mutation and its composition of inserted (+) and deleted (Δ) sequence is shown to the right of the sequence as well as the number of times a sequence appeared.

We compared the ability of our *trans*-splicing CRISPR/Cas9 system (tsSpCas9) with wild-type SpCas9 (wtSpCas9) in cleaving DNA sequences at different loci in different human and mouse cell lines. Cleavage by tsSpCas9 paired with appropriate sgRNAs targeting the well-studied hemoglobin beta (*HBB*) and C-C chemokine receptor type 5 (*CCR5*) genes^24^ (Fig. 3a) was tested by plasmid transfection in human HEK-293T cells. Specifically, each of the two tsSpCas9 plasmids (containing expression cassettes for the sgRNAs as well as one of the tsSpCas9 pieces, Fig. 1c) was delivered at half of the dose of the wtSpCas9 plasmid so that the total mass of tsSpCas9 plasmids matched the total mass of wtSpCas9 plasmids, and the mutations induced in 293T cells were measured using the T7 Endonuclease I (T7E1) assay. We found that tsSpCas9 induced mutations at the *HBB* locus with a frequency of 18 ± 5%, while the frequency induced by wtSpCas9 was 36 ± 12% (mean ± standard deviation), indicating that the trans-splicing system was achieving roughly half the mutation rate of the wild-type SpCas9 (Table 1). Selected samples from the wtSpCas9 and tsSpCas9 transfections were also analyzed by TOPO sequencing which showed the characteristic insertions and deletions caused by NHEJ (Fig. 3c). Two sets of offset guide RNAs paired with D10A “nickase” SpCas9 (paired nickases)^1^ were also tested at *HBB* (Fig. 3b) and the tsSpCas9 systems achieved ~30% of the activity of the corresponding wtSpCas9 paired nickase systems (Table 1). At *CCR5*, tsSpCas9 induced mutations in 11 ± 1% of alleles while wtSpCas9 had a frequency of 38 ± 4%, indicating that at this locus the trans-splicing system achieved ~29% of the activity of the wild-type SpCas9 (Table 1). Control transfections demonstrated that all components of the tsSpCas9 system were necessary to induce DNA cleavage (Supplementary Figure S3).

It is likely that the kinetics of the tsSpCas9 system are altered compared to wtSpCas9 due to the requirement that trans-splicing has to occur. We therefore examined if there are differential dose-dependent effects on nuclease activity between tsSpCas9 and wtSpCas9. Transfection of 293T cells with varying plasmid doses of either tsSpCas9 or wtSpCas9 targeting *HBB* and *CCR5* showed that there is a strong dose dependent effect for tsSpCas9. Over a range of decreasing doses over an 8-fold range, wtSpCas9 decreased in activity as previously reported^24^, but the tsSpCas9 had a much shaper decline in activity at the lower doses than wtSpCas9 (Table 1), which was consistent with our hypothesis that the kinetics of the multi-component tsSpCas9 system lead to a stronger dose-dependent decrease in activity.

### Testing *trans*-splicing SpCas9 in additional cell types

**Figure 4:**
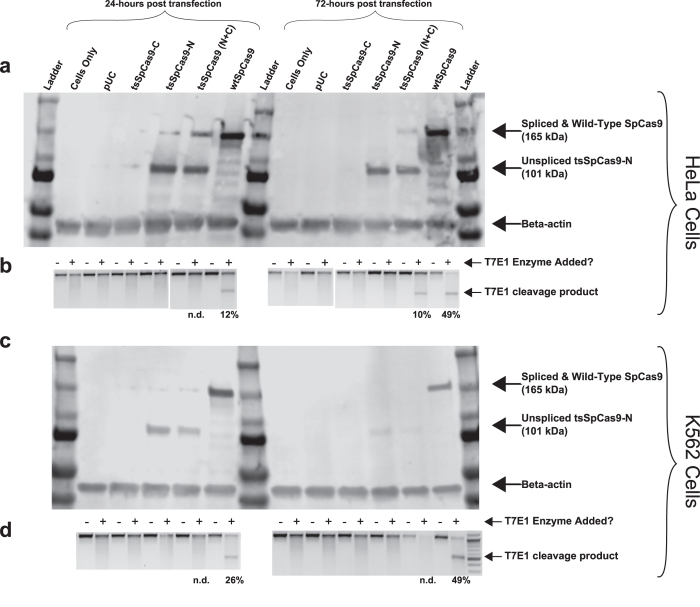
tsSpCas9 splicing and nuclease activity in additional cell lines. (**a** and **c**) Western blotting for the 3xFLAG epitope present on tsSpCas9-N and wtSpCas9 at 24 hours and 72 hours post-transfection in HeLa and K562 cells respectively. (**b** and **d**) T7E1 analysis of nuclease activity in HeLa and K562 cells respectively. Percent mutated alleles listed below gel lanes or n.d. for not detected.

Although the tsSpCas9 system worked robustly in human HEK-293T cells, in human K562, U87, and U2OS cell lines as well as mouse NIH-3T3 cells, the tsSpCas9 system did not induce detectable levels of non-homologous end-joining (NHEJ) despite robust activity (20%-50% allele modification frequency) by wtSpCas9 (Fig. 4d for K562 cells, data not shown for other cells types). In HeLa cells, detectable protein splicing (Fig. 4a) and nuclease activity (Fig. 4b) was observed with the tsSpCas9 system, but attempts to replicate the finding in 24-well plate format (experiments for Fig. 4 were in larger 6-well plates) failed to detect any tsSpCas9 activity. For unknown reasons, the transfection efficiency of HeLa cells was markedly lower (20% GFP+ vs. 90% GFP+) in 24-well plate format compared to the 6-well plate format; since tsSpCas9 requires successful co-transfection of two plasmids for nuclease activity, the lower transfection efficiency may explain the lack of detectable nuclease activity. Further attempts to replicate the results in 6-well plate format failed to achieve high transfection efficiencies (<40% GFP+) for unknown reasons. Low transfection efficiency (~20% GFP+) in NIH-3T3 cells may have contributed to the lack of tsSpCas9 activity in those cells. However we do not suspect transfection efficiency is a major contributor to the failure of tsSpCas9 in K562, U87, or U2OS cells because the %GFP+ cells was robust between 60-80%. Western blotting in K562 and HeLa cells showed that tsSpCas9-N was expressed in both cell types but splicing was relatively inefficient, with the majority of the tsSpCas9-N remaining in the unspliced (lower molecular weight) form (Fig. 4a, **c**), although faint bands of the spliced tsSpCas9 can be detected at the 24 hour timepoints in both cell types and at 72 hours post transfection in HeLa cells. Previous work with the GyrA split intein has suggested that fusing the FKBP fragment to the C-terminus of the split fragment could help improve solubility and splicing^17^; the length of the fusion is relatively short (~330 bp) and so would not appreciably decrease the remaining space within the theoretical AAV packaging limit for the tsSpCas9 system.

Analysis of protein levels and nuclease activity at two timepoints (24 hours and 72 hours post-transfection) revealed in all three cell types examined (293T in Fig. 1e, HeLa in Fig. 4a, and K562 in Fig. 4c) that tsSpCas9 protein levels (and to a lesser extent for wtSpCas9) appeared higher at 24 hours than 72 hours post-transfection. Mutated alleles increased over time as would be expected from cumulative nuclease activity inducing double-strand breaks and subsequent mutagenic repair by NHEJ.

## Discussion

Due to the large size of Cas9 and dCas9 fusion proteins, their *in vivo* delivery via AAVs remains challenging. As a first step in developing a strategy that offers flexibility in promoter choice to package Cas9 and the associated effector domains in an AAV vector, we successfully demonstrated that trans-spliced Cas9 protein allows cleavage of *HBB* and *CCR5* genes in human HEK-293T cells with good efficiency. Our results indicate that intein-mediated protein trans-splicing offers great potential to circumvent the AAV packaging limitations to allow ample space for longer genetic elements such as dCas9 fusion proteins, tissue specific promoters, and expression of multiple sgRNAs. Compared to recent findings by Wright *et al*.^25^ of an alternate method of splitting SpCas9, our system appears to be much more efficient at inducing mutations in human HEK-293T cells; while our tsSpCas9 system experienced a ~3 fold reduction in activity compared to wtSpCas9, the system described by Wright *et al*. experienced a >30 fold reduction in activity.

The GyrA intein inserted into SpCas9 at amino acid position 656 allows for nuclease activity in cells after the protein is expressed from two separate cassettes of lengths well within the packaging limit of AAVs. While this intein system did not work in several cell types tested, it is possible that other inteins, fusion of solubility enhancers, or alternate split points within SpCas9 or other Cas9 orthologs may allow for systems which better facilitate flexibility in promoter choice and dCas9 fusions when delivering CRISPR/Cas9 systems using AAVs. Furthermore, while standard dosing levels of the tsSpCas9 system resulted in relatively comparable levels of nuclease activity compared with wtSpCas9 (2-3 fold reduction in activity), the tsSpCas9 had much lower activity than that of wtSpCas9 at low doses. It remains to be determined what dose levels can be effectively achieved *in vivo* at different organs with AAV delivery of SpCas9 and whether the reduction in tsSpCas9 activity would be an issue.

## Methods

### Plasmid Construction

Mammalian codon optimized versions of the split inteins were synthesized by GenScript and cloned into the pX330 vector^2^ using restriction enzymes. For the tsSpCas9-GyrA-N plasmids, the enzymes EcoRI and EcoRV were used. For the tsSpCas9-Gyra-C plasmids, the enzymes AgeI and PmlI were used. Restriction enzymes were ordered from New England BioLabs. The 5’ ITR was cloned into the pX330 and pX335 plasmid backbones as two annealed oligo sequences after digesting the plasmids with AflIII. The plasmids for the tsSpCas9 system using the Mxe GyrA intein are available on AddGene through the Gang Bao Lab webpage (http://www.addgene.org/gang_bao/): tsCas9-N (#58693), tsCas9-C (#58694), and tsCas9-Nick (#58695). Full open reading frame and plasmid sequences are provided in Supplemental Data D1 and D2. Guide strands were cloned into the backbones using the BbsI enzyme exactly as previously described^2^. Plasmids for cellular experiments were prepped using the EndoFree Plasmid Maxi Kit (Qiagen, #12362).

### Cell culture and transfection

For all plasmid delivery, each of the two tsSpCas9 plasmids was delivered at half of the dose of the wtSpCas9 plasmid so that the total mass of tsSpCas9 plasmids matched the total mass of wtSpCas9 plasmids. This approach was chosen to examine a “practical use” scenario for applications where the total amount of DNA is a limiting factor (i.e. before inducing cell toxicity or immune response to AAVs) rather than one that more rigorously examines trans-splicing kinetics where equal amounts of wtSpCas9 and tsSpCas9 protein would be expressed. Experiments with delivery of only a single tsSpCas9 plasmid were also performed at the half dose amount, supplemented with pUC plasmid to normalize the total mass of plasmid delivered.

HEK-293T cells (ATCC) were cultured in DMEM supplemented with 10% FBS and 2 mM L-glutamine. For transfections, 6e4 cells/well in 500 uL of media were seeded into 24-well plates coated with 0.1% gelatin 24 hours prior to transfection. Triplicate transfection mixes were made containing 660 ng of experimental plasmid (various ratios of nuclease and pUC) and 6.6 ng of eGFP plasmid in 14.4 uL of nuclease-free water. 10.2 uL of FuGene HD (Promega) was added to each triplicate mix, allowed to incubate for 5 minutes, and then 25 uL of that mixture was pipetted into each well, corresponding to 1000 ng of experimental plasmid and 10 ng of eGFP delivered to each well (the maximum dose of 1000 ng was based on the highest Cas9 activity achieved in our previously reported results^24^). Media was replaced 48 hours after transfection. 72 hours after transfection, media was aspirated, and 20 uL of Trypsin-EDTA was added to each well to detach the cells. Trypsin was neutralized by adding 150 uL of PBS supplemented with 2% FBS. Samples of each transfection were run through an Accuri C6 flow cytometer to quantify transfection efficiency via the percent of GFP+ cells using a 510 ± 15 nm emission filter. Transfection efficiencies were roughly similar between all 293T experiments (~80% GFP+); therefore slight variations in transfection efficiency were included as a component of the standard deviation between replicates rather than normalizing nuclease efficiencies by transfection efficiency. All cells were pelleted (cells were not sorted for GFP fluorescence) by centrifugation at 300 g for 5 minutes. The supernatant was aspirated and 80 uL of QuickExtract solution was added for DNA extraction according to the manufacturer’s protocol (EpiCentre). The ½ dose experiments corresponded to 500 ng of Cas9 plasmid and 500 ng of pUC (1000 ng total); ¼ dose contained 250 ng of Cas9 and 750 ng pUC; 1/8 dose contained 125 ng of Cas9 and 875 ng pUC.

### Western Blotting

Larger transfections were performed to obtain sufficient sample for Western blotting. Transfections were similar to those described above with the following modifications. For 293T cells, all reagent doses (i.e. FuGene, cell seeding, DNA doses) were scaled up 5-fold for use in 6-well plates. HeLa cells (ATCC) were transfected similar to 293T cells with the following modifications (determined by previous transfection optimization in our laboratory of CRISPR/Cas9 in HeLa cells), 1.8e5 cells/well were seeded in 6-well plates, wells were not coated with gelatin, only 3000 ng total DNA was used, and less FuGene (58% of the amount used for 293T cells) was added. For each condition for 293T and HeLa cells, three wells of a 6-well plate were transfected; two were harvested 24 hours post-transfection and combined for downstream analysis while the remaining well was cultured for 72 hours post-transfection before harvesting. K562 cells (ATCC) were transfected using the 4D Nucleofector system (Lonza) according to the manufacturer’s instructions. Briefly, 1e6 cells were resuspended in 100 uL of “Lonza SF” solution, 10 uL of DNA at 500 ng/uL (5000 ng total) was mixed in, the cuvette was pulsed with the “FF-120” sequence, and cells were transferred into 2.5 mL of pre-warmed RPMI-1640 media (Life Technologies) containing 10% FBS and L-Glutamine in a 6-well plate. For each condition, three wells of a 6-well plate were transfected; at 24-hours post-transfection all three wells were combined (K562 cells grow in suspension), 1e6 cells were harvested for analysis, and the remaining cells were re-plated and cultured until 72 hours post-transfection before harvesting.

Cells were harvested using 50 μl of RIPA lysis (Peirce Biotechnology) supplemented with the HALT protease cocktail (Peirce Biotechnology, without EDTA). To quantify protein levels a BCA assay was performed (Peirce Biotechnology microBCA kit). A volume was calculated such that 10 μg of protein would be loaded into each well. Lysate was diluted into a mixture of 4x Laemmli buffer and water, heated for 10 minutes at 100 ^o^C, and subsequently loaded onto 15 well 4-15% mini protein TGX gels (BioRAD). 10 ul of BioRAD precision plus protein standard was used as a ladder (10 – 250 kDA range). The gel was run for 45 minutes at 130 V. Transfer onto a PVDF membrane was performed (0.45 um pore size) at 4 ^o^C overnight (~16 hours) at 10 V in a transfer buffer containing 10% methanol. Blocking was performed for 1 hour at room temp on a shaker in 10 ml Rockland blocking buffer supplemented with 3% donkey serum (Jackson Scientific). Primary antibodies for actin (mouse monoclonal 1:1000 dilution, mABGEa, Peirce) and anti-flag (monoclonal mouse M2, Sigma-Aldrich, 1:5000) were incubated overnight at 4 ^o^C in Rockland blocking buffer supplemented with 0.1% Tween-20. After TBS-T washes, the secondary antibody (Licor 680 donkey α-mouse, 1:15000) was added in Rockland blocking buffer supplemented with 0.1% Tween-20 and 0.01% SDS and allowed to incubate for 1 hour on a shaker at room temperature. After final TBS-T washes, membranes were stored in TBS and imaged using the LICOR Odyssey scanning system.

### Nuclease Activity Assays

50 uL PCR reactions were performed using 0.2 uL of AccuPrime Taq HiFi (Invitrogen), AccuPrime Buffer2, 1 uL of QuickExtract genomic DNA, and 0.2 uM of forward and reverse primers. Thermocycler conditions were: 2 minutes of 94 °C denaturation, 40 cycles of 30 seconds of 94 °C melting, 30 seconds of annealing at 60 °C, and 60 seconds of 68 °C extension, followed by a final 10 minute extension at 68 °C. Primer sequences are listed in Supplementary Methods M1. PCR reactions were purified using EZ-Pure magnetic beads (Omega Biotek) and eluted in nuclease-free water. T7 Endonuclease I (T7E1) assays were performed exactly as previously described^26^. TOPO cloning was performed using the Zero Blunt kit (Invitrogen) according to manufacturer’s instructions and sequenced using the M13 forward sequencing primer.

## Additional Information

**How to cite this article**: Fine, E. J. *et al*. Trans-spliced Cas9 allows cleavage of *HBB* and *CCR5* genes in human cells using compact expression cassettes. *Sci. Rep*. **5**, 10777; doi: 10.1038/srep10777 (2015).

## Supplementary Material

Supplementary Information

## Figures and Tables

**Table 1:** Comparison of tsSpCas9 to wtSpCas9 gene modification activity.

		Nuclease / Nickase		% retained activity	% modified alleles	
Gene	Guide RNA(s)		Dose		tsSpCas9	wtSpCas9
HBB	R-2 / R-1	Nickase	Full	31.1%	17.3 ± 4.1%	55.6 ± 19.9%
	R-2 / R-7			25.2%	19.0 ± 4.0%	75.3 ± 1.1%
	R-3	Nuclease	Full	49.2%	17.8 ± 5.5%	36.2 ± 12.0%
			1/2	50.6%	18.6 ± 0.6%	36.7 ± 11.0%
			1/4	27.9%	8.2 ± 2.3%	29.6 ± 14.4%
			1/8	8.9%	2.0 ± 1.4%	22.3 ± 16.0%
CCR5	R-30		Full	29.2%	10.9 ± 1.2%	37.5 ± 4.2%
			1/2	18.6%	5.8 ± 1.0%	31.0 ± 2.5%
			1/4	14.5%	3.4 ± 0.3%	23.2 ± 1.7%
			1/8	5.1%	0.9 ± 0.4%	18.7 ± 2.8%

Human HEK-293T cells were treated with plasmids encoding either wtSpCas9 or tsSpCas9. Percent retained activity refers to the ratio of modified alleles in tsSpCas9-treated cells compared to wtSpCas9-treated cells. Modified alleles are mean ± s.t.d. (n = 3 for most samples, n = 2 for ‘R-3 wtSpCas9 Full Dose’ and ‘R-3 tsSpCas9 1/8 Dose’).
